# Circadian and Metabolic Effects of Light: Implications in Weight Homeostasis and Health

**DOI:** 10.3389/fneur.2017.00558

**Published:** 2017-10-19

**Authors:** Santiago A. Plano, Leandro P. Casiraghi, Paula García Moro, Natalia Paladino, Diego A. Golombek, Juan J. Chiesa

**Affiliations:** ^1^Chronophysiology Laboratory, Institute for Biomedical Research (BIOMED – CONICET), School of Medical Sciences, Universidad Católica Argentina (UCA), Buenos Aires, Argentina; ^2^Laboratorio de Cronobiología, Universidad Nacional de Quilmes – CONICET, Buenos Aires, Argentina

**Keywords:** photic entrainment, suprachiasmatic nucleus, metabolism, desynchronization, obesity

## Abstract

Daily interactions between the hypothalamic circadian clock at the suprachiasmatic nucleus (SCN) and peripheral circadian oscillators regulate physiology and metabolism to set temporal variations in homeostatic regulation. Phase coherence of these circadian oscillators is achieved by the entrainment of the SCN to the environmental 24-h light:dark (LD) cycle, coupled through downstream neural, neuroendocrine, and autonomic outputs. The SCN coordinate activity and feeding rhythms, thus setting the timing of food intake, energy expenditure, thermogenesis, and active and basal metabolism. In this work, we will discuss evidences exploring the impact of different photic entrainment conditions on energy metabolism. The steady-state interaction between the LD cycle and the SCN is essential for health and wellbeing, as its chronic misalignment disrupts the circadian organization at different levels. For instance, in nocturnal rodents, non-24 h protocols (i.e., LD cycles of different durations, or chronic jet-lag simulations) might generate forced desynchronization of oscillators from the behavioral to the metabolic level. Even seemingly subtle photic manipulations, as the exposure to a “dim light” scotophase, might lead to similar alterations. The daily amount of light integrated by the clock (i.e., the photophase duration) strongly regulates energy metabolism in photoperiodic species. Removing LD cycles under either constant light or darkness, which are routine protocols in chronobiology, can also affect metabolism, and the same happens with disrupted LD cycles (like shiftwork of jetlag) and artificial light at night in humans. A profound knowledge of the photic and metabolic inputs to the clock, as well as its endocrine and autonomic outputs to peripheral oscillators driving energy metabolism, will help us to understand and alleviate circadian health alterations including cardiometabolic diseases, diabetes, and obesity.

## Introduction

The 24-h transitions between light and darkness [light:dark (LD) cycles] generated by the rotation of the Earth provide a strong temporal cue (i.e., the time giver, or *zeitgeber*) for most organisms, which display daily behavioral and physiological rhythms. In mammals, a hierarchical coordination is exerted from a central circadian clock at the suprachiasmatic nucleus (SCN) of the hypothalamus. SCN activity orchestrates clocks in peripheral vegetative functions by endocrine and autonomic outputs [reviewed in Ref. (1, 2)], in resonance with the cyclic environmental changes. Notably, such changes affect not only core body temperature (CBT), hormonal secretion, or the timing of sleep but also the homeostatic balance of nutrients intake, processing, and energy expenditure. Indeed, most mammalian genes oscillate in cell-autonomous clocks that interact directly or indirectly with feeding-fasting cycles (3). This helps to keep daily rhythms in tissue functions, setting adequate timing for nutrient assimilation, mobilization and distribution, and metabolic waste removal. This center-to-periphery orchestration is phase stabilized by LD phototransduction at the SCN (4). A fine-tuning of this process is exerted by peripheral feedback into the SCN through behavioral, neural, and/or humoral factors, while peripheral oscillators receive entraining signals directly from systemic blood-borne metabolites (2).

Recent studies revealed that circadian disruption of this network at different levels might lead to metabolic and weight homeostasis alterations. Under normal light entraining conditions, adequate phase relationships between the LD cycle and body rhythms (e.g., activity/rest, feeding/fasting rhythms, and metabolic hormones) are achieved, even when taking into account short-term acute variations induced by exercise or feeding. Indeed, the timing of behavioral activity and peripheral rhythms is separated through endocrine and autonomic outputs ensuring a day–night variation in cardiometabolic drive (5). This also applies to metabolic rhythms that are needed to sustain and even prepare the body for adequate alertness. For instance, basal glycemia is daily regulated by insulin, glucagon, and growth hormone rhythms, while glucocorticoids regulate both glucose uptake and catabolism in muscles, brain, and adipose tissue for awakening and activity onset (6).

This article will review the role of photic entraining conditions in circadian oscillation, metabolism and energy expenditure, and body weight homeostasis, considering the lighting conditions commonly used in the laboratory for animal’s studies (i.e., LD cycles, photoperiod length, constant light and darkness), as well as light pollution in urban environments, helping to understand its implications for human health alterations leading to metabolic syndrome (MS) and obesity.

## The Circadian System

### The SCN Clockwork

The circadian clock is located above the suprachiasmatic tract, receiving light information from the retina to entrain the SCN activity with LD cycles. The SCN is formed by two bilateral nuclei of ~20,000 neurons distributed into two major subregions: a ventrolateral region, receiving most retinal afferents expressing vasoactive intestinal peptide (VIP), and gamma-aminobutyric acid (GABA), and a dorsomedial region expressing arginine-vasopresin peptide, GABA, and receiving/projecting afferents/efferents from/to other hypothalamic nuclei (7). Most of the dorsomedial neurons exhibit circadian electrical activity through self-sustained transcription-translation feedback loops (TTFL) involving clock-gene expression and its post-translational regulation (8). The transcriptional factor CLOCK and ARNTL, and ARNTL2 form heterodimers which binds to E-boxes in the promoters of their own repressors, Period (*Per1-3*) and Cryptochrome (*Cry1-2*), and of the nuclear hormone receptors Rev-erb (α and β), and *Ror* (α, β, and γ). Two additional loops control *Bmal1* expression, by REV-ERBα repression, and RORα activation through RRE elements in its promoter (9). The TTFL molecular machinery is functional in almost all cells in peripheral tissues and is driven by direct autonomic and/or endocrine outputs of the SCN, as well as by indirect blood-borne metabolites temporally gated by the SCN (3).

### Photic Entrainment of the Circadian Clock

Clock-controlled mechanisms allow anticipatory temporal organization of biological functions according to predictable changes (e.g., the daily LD cycle) and, just as importantly, a temporal segregation between conflicting or incompatible processes (e.g., feeding and sleeping). Thus, daily photic entrainment of the SCN clock, i.e., to adjust its period with that of the LD cycle, is a key organizer for such temporal homeostasis. Light stimuli synchronize the SCN through glutamatergic signaling by means of the retinohypothalamic tract (4). Ventrolateral neurons respond to photic stimulation during the subjective night in nocturnal rodents by increasing the expression of clock genes of the Period (Per) family, per1 and per2 (10–13). In turn, signaling from the retinorecipient ventrolateral neurons to the dorsomedial SCN synchronize self-sustained clock-gene oscillations. Rhythmic intercellular coupling allows the SCN to act as a tissue pacemaker with coherent circadian outputs. Such coupling occurs by neuropeptide signaling, such as VIP (through VPAC receptors), GRP-mediated communication (14, 15), and NO and GABA neurotransmission (16–20). Uncoupling the SCN neuronal network of gene and neurotransmitter activity by untimed circadian lighting generates dampened SCN outputs controlling behavior and physiology. The SCN acts as the hypothalamic link between the retina and oscillators in peripheral organs, entraining those organs to the LD cycle.

### SCN Outputs That Temporize Tissue Oscillators

**Figure 1:**
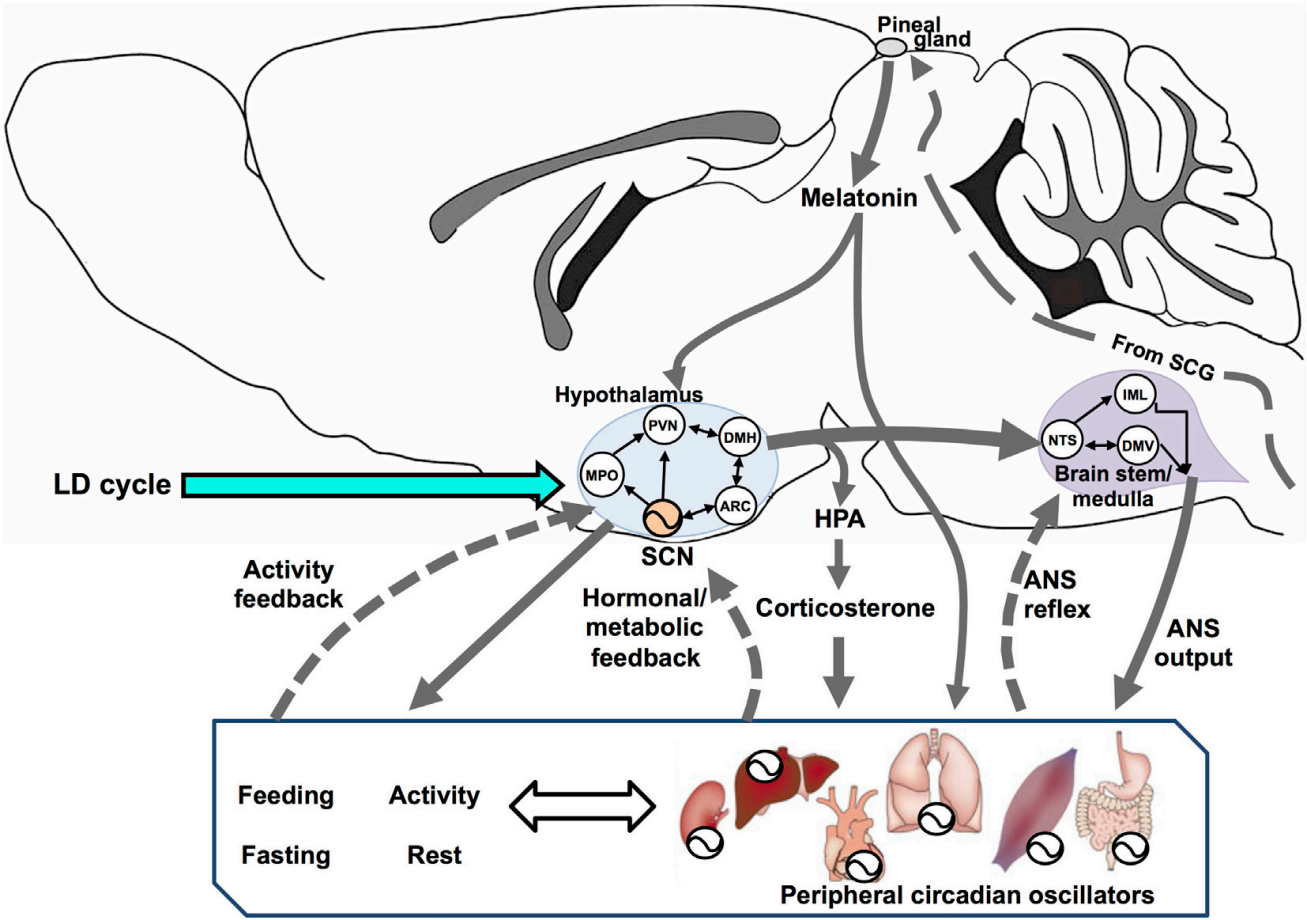
Circadian rhythms are driven by a vast network of oscillators regulated by multiple interconnected feedback loops that, in turn, synchronize the entire organism. Here, we show a simplified system with three interconnected components: the hypothalamus, brain stem/spinal cord, and the periphery (including behavioral rhythms as feeding/fasting and activity/rest). In the hypothalamus, the suprachiasmatic nucleus (SCN) sends synchronizing signals to different hypothalamic areas such as the medial preoptic (MPO), paraventricular (PVN), dorsomedial (DMH), and the arcuate (ARC) nuclei. All these are interconnected and send feedback information to the SCN. In addition, PVN efferences connect to two main endocrine outputs: (1) a polysynaptic pathway relaying in the superior cervical ganglion (SCG) which controls the production and release of melatonin from the pineal gland *via* its sympathetic innervation and (2) the secretion of corticotrophin-releasing hormone, acting on the pituitary for the release of adrenocorticotropic hormone (HPA axis) and controlling adrenal glucocorticoids (corticosterone in rodents). The SCN exert a circadian control on PVN outputs for most autonomic nervous system (ANS) functions, driven at the brain stem/medulla through parasympathetic motoneurons in the vagal dorsal motor nucleus (DMV), and by sympathetic motoneurons in the intermediolateral column (IML). The nucleus tractus solitarius (NTS) acts as an integrative center for signals coming from the hypothalamus, peripheral ANS reflexes transmitted to the DMN and the IML, and feedback to the hypothalamus (not shown). Blood-borne factors like glucose, feeding/fasting regulatory hormones and, factors derived from physical exercise, can modulate circadian rhythms at peripheral organs, as well as regulate the ANS feedback to the hypothalamus.

The SCN is a central timing structure that organizes circadian oscillators controlling metabolism, primarily by maintaining behavioral rhythms of activity/rest and feeding/fasting, through both synaptic (21) and diffusible factors (22) that couple the SCN with neuroendocrine and neural outputs. SCN neurons exhibit an endogenous rhythm of electrical activity linked to the molecular oscillator (23), driving rhythmic neurotransmitter release at their efferents (24) even under constant dark conditions (25). Many brain areas serve as effectors of the circadian clock, including projections to different targets such as the paraventricular nucleus of the hypothalamus (PVN) and other non-hypothalamic areas that gate physiologically relevant sensory information (26). One of the major outputs of the SCN is directed toward the control of hormonal secretions, by means of (1) direct contact with neuroendocrine neurons at the PVN, controlling the release of corticotropin-releasing hormone (CRH) and stimulating the adenohypophysis for the release of adrenocorticotropic hormone (ACTH); (2) targeting intermediate neurons, e.g., neurons of the medial preoptic nucleus, the dorsomedial hypothalamic nucleus (DMH), or the sub-PVN; (3) stimulating autonomic PVN projections to intervene into autonomic nervous system (ANS) circuits that affects endocrine organs; and finally (4) by influencing its own feedback (24). In addition, CBT rhythm is a relevant SCN output which can temporize peripheral tissues (27). Figure 1 outlines the integration of circadian energy metabolism, by the main brain structures regulating behavioral, endocrine, and physiological outputs, and the main pathways for peripheral interactions and feedbacks.

The SCN controls several outputs driving diurnal rhythms in metabolic hormones to regulate energy homeostasis. A main SCN–CRH–ACTH output drives a rise in adrenal glucocorticoids just before activity onset. This promotes arousal and alertness by enhancing liver gluconeogenesis (from amino acids and fatty acids), promoting release of liver glucose to the blood, and increasing its uptake in the brain and muscles. Adrenal glucocorticoids have been implicated as a peripheral humoral cue for the entrainment of oscillators such as the liver (28). Glucocorticoids can also feedback to either the SCN or its outputs driving behavioral re-entrainment to light in a model of jet-lag (29). The systemic levels of the main metabolic hormones, insulin, glucagon, and adrenalin also exhibit robust circadian rhythms controlled by the SCN (1). The circadian rhythm of glucose has a direct drive from the SCN through a sympathetic pathway that connects with the liver (30). The SCN increases insulin sensitivity through multiple pathways, resulting in an increase in glucose uptake in muscle, and simultaneously causes increased hepatic glucose production (31, 32). In addition, the SCN exerts a direct control on cardiac and glucose metabolism (24). Circadian and LD-controlled pineal melatonin release is driven by SCN output through a multisynaptic pathway relaying in the sympathetic superior cervical ganglion (33, 34), regulating metabolism in photoperiodic species (see below).

### Entrainment of Peripheral Oscillators

Peripheral and central clocks are normally held in adaptive synchrony by the SCN. In particular, the central clock regulates rhythmic feeding behavior, which in turn produces rhythmic food intake generating circadian-metabolic signals in the periphery. Endocrine and autonomic SCN outputs to peripheral tissues regulating nutrients intake, processing, mobilization, usage for fuel and/or storage provide a circadian control on global energy metabolism. While the circadian activity/rest rhythm is entrained to the LD cycle, the feeding/fasting rhythm appears to affect the timing and robustness of circadian rhythms in metabolic organs. As a general rule, peripheral oscillators (like the liver) respond rapidly to cycles of food availability, while the light cycle resets the SCN more quickly than does food (35, 36). Restricting food intake during the light, resting phase in nocturnal rodents under LD, in addition to affect CBT and glucocorticoid amplitudes, completely reverses clock-gene expression in the liver, kidney, heart, and pancreas, but do not affect SCN function (37). Indeed, the expression of genes controlling peripheral clocks can be regulated directly by the circadian clock, by systemic signals from periodic food ingestion, or by a combination of both mechanisms (38, 39). While the circadian component of this regulation optimizes the anticipation of predictable fluctuations in the organ environment, the responsiveness to systemic cues adds a degree of flexibility to the system that is necessary to adapt to unpredictable changes in the environment (e.g., in times of caloric intake restriction, and/or temporal niche switching, see below).

Peripheral clocks are strongly affected by cell metabolism, the physiological and metabolic state of the surrounding tissue, and serum-borne signals. Several metabolic signals such as NADH/NAD+, NADPH/NADP+, ATP/AMP, oxygen and its reactive species, carbon monoxide, or glucose, directly affect transcriptional activity of circadian proteins (40). Peripheral oscillators such as the liver clock are additionally coupled to cellular and general physiology through energy and nutrient sensing systems, such as AMP-activated protein kinase (AMPK), PPARGC1A (also known as PGC1), metabolic feedback loops involving metabolites, such as polyamines, and nuclear hormone receptor signaling pathways.

Peripheral oscillators not only adjust their clock-genes rhythms to the master circadian clock by a mix of direct and indirect signals from the SCN but also can be differentially altered in phase by behavior (e.g., activity, or access to food) or light. While the liver clock adjusts its phase preferentially to feeding time, adrenal glands strongly respond to light input Ishida (41, 42). Animals receiving food six times a day lose their rhythm in white adipose tissue in seven of nine tested oscillatory metabolic/adipokine genes, but not in clock genes. By contrast, abolishing the daily corticosterone peak also rendered the peripheral clock-genes arrhythmic (43).

Multiple circadian clocks drive local rhythms of cellular metabolism, synchronized by cues from the SCN as well as by other inputs. Autonomous rhythms in the mediobasal hypothalamus might contribute to metabolic homeostasis (44–49). Moreover, the DMH might play an important role as a component of the SCN-independent food entrainable oscillator (50–53). Importantly, regulation of body weight involves the integration of multiple signals in a neural network of several interconnected hypothalamic areas, including the SCN, the arcuate nucleus (AN), the ventromedial hypothalamic nucleus (VMH), and the PVN, whose interactions control the main three factors that underlie energy balance: appetite and food intake, deposition of fat, and energy expenditure (2, 54).

### Circadian and Metabolic Integrative Function in the Liver

The functions of the liver are rhythmically regulated; an analysis of transcriptions within the liver shows two peaks corresponding to the dusk and dawn oscillators, and this may reflect the differences in physiological requirements such as energy demand (55–58). The liver orchestrates rhythms in energy homeostasis, i.e., levels of glucose transporters, the glucagon receptor, and enzymes regulating the metabolism of hexose sugars were observed with peak phase of expression in accordance with the rhythm of feeding. In addition, enzymes related to lipid homeostasis such as glycerol 3-phosphate pathway (e.g., glycerol-3-phosphate acyltransferase, 1-acyl-glycerol-3-phosphate acyl-transferase, and lipins), which regulates glycerol and lipid metabolism and triglyceride accumulation, are expressed in a circadian manner (59).

The liver circadian clock acts by buffering the fluctuations of blood glucose levels originated by behavioral and feeding/fasting cycles in a circadian fashion. Specific Bmal1 deletion in the liver reduced basal glycemia in mice during the fasting phase, mainly by altering liver glucose export (60), insulin and glucagon secretion (61, 62), and glucose production and uptake (32, 63). In addition, many clock-genes and clock-controlled genes have multiple roles in glucose metabolism. Cryptochromes regulate hepatic gluconeogenesis through interaction with G protein-coupled receptors, inhibiting cAMP storage and increasing transcription of CREB-regulated gluconeogenic genes. In addition, overexpression of Cry1 in liver affects glucose levels in diabetic mice (64). Positive and negative regulators of the core clock oscillator control circadian regulation of the glucose metabolism. Multiple signaling pathways converge on rate-limiting enzymes of glucose anabolism and catabolism, such as phosphoenolpyruvate carboxykinase or pyruvate kinase (65). As mentioned earlier, the liver oscillator is strongly coupled to feeding/fasting cycles, and its insulin-dependent fluctuations, in addition to being regulated at the level of Per1-2 genes (66).

## Lighting Conditions and Their Impact on Metabolism and Weight Homeostasis in Animal Models

Murine models have long been the first choice to study the effects of environmental circadian challenges. Lighting conditions in the laboratory can be manipulated through many protocols: adjusting the LD period (i.e., generating T-cycles), determining the relative duration of light and dark intervals, regulating light intensity, applying light- or darkness-pulses, or maintaining constant routines of darkness or light. The possibility of combining all of these should also be considered, thus providing a myriad of different challenging lighting environments.

12:12 h “square” LD cycles with abrupt light–dark transitions constitute a “standard” condition in most chronobiology studies with rodents. However, it is difficult to state, from the metabolic point of view, which is the “normal” (i.e., eco-physiological) lighting condition, since it might be quite variable in the daily and/or annual photoperiodic environment. In addition, intrinsic photic features of the laboratory LD cycles, such as light intensity (i.e., the number of light hours per day, or photoperiod length), can modulate behavioral entrainment, metabolism, and weight. Spectral composition, abrupt or twilight transitions, and the irradiance contrast between light and dark portions of the LD cycle should also be considered. Several laboratories have designed “dim light at night” protocols to simulate night light pollution in urban environments, which will be discussed below (67).

### Laboratory 24-h LD Cycles

#### LD Cycles and Temporal Niche Interaction

**Figure 2:**
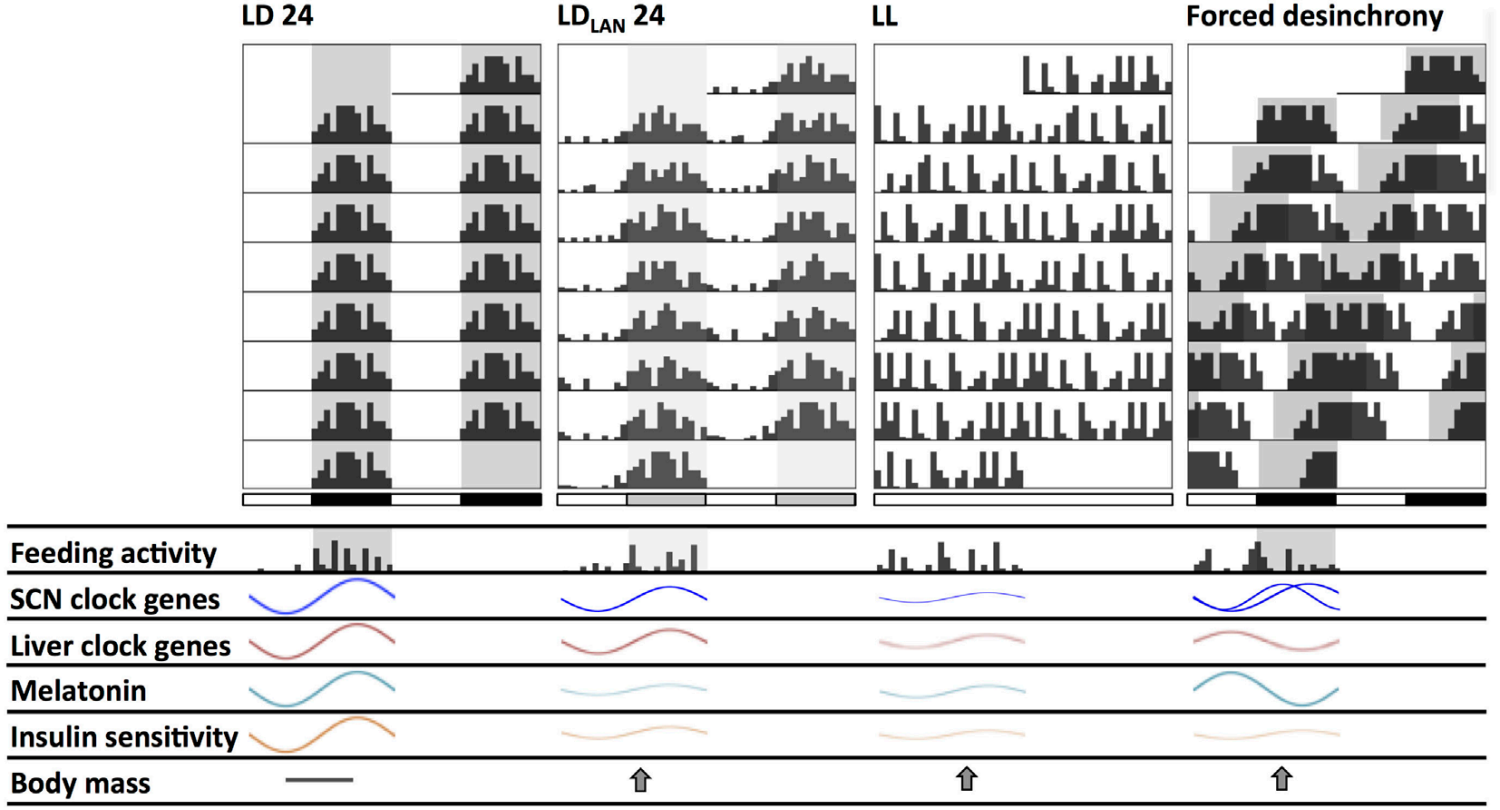
Schematic changes found in mice and rats chronically exposed to different lighting protocols which might induce circadian and metabolic alterations. Actograms double plotted at modulo 24 h show alterations in the behavioral activity rhythm. Compared to standard light:dark (LD) conditions: (1) LD_LAN_ (light at night) promotes a dispersed rhythm increasing both general and feeding activity bouts at the light phase, together with reduced suprachiasmatic nucleus (SCN) and liver clock-genes amplitude; (2) LL generates behavioral arrhythmicity with loss of the feeding/fasting rhythm, also with dampened amplitude of SCN and liver clock-genes rhythms; (3) forced desynchrony protocols (i.e., chronic jetlag—CJL—and T cycles) generate two activity components at the behavioral and SCN clock-gene (regional) levels, with disrupted daily feeding/fasting rhythms, and with liver clock genes out of phase. Dampened melatonin rhythms occur both under LL and LD_LAN_, while this rhythm is out of phase under forced desynchronization. All lighting protocols promote a decrease in the insulin sensitivity rhythm, and an increased weight gain respect to LD.

Besides circadian photoentrainment, and the subsequent interest in peripheral oscillators, it is interesting to consider the temporal niche of the species and the adaptive plasticity of the circadian system (68). This is a major hallmark for present and future chronobiology, since it includes the comprehension of the center-to-periphery interactions among circadian oscillators, as well as their eco-physiological interactions with environmental cycles. Indeed, temporal niche variations (i.e., “switching”) is a strategy used in several rodents during their circannual or life cycle (69). In addition, most of the chronobiological studies utilize nocturnal rodents, and not all conclusions may be applied to understand metabolic alterations in humans, a typical diurnal mammal. Although activity/rest rhythms are 180° out of phase, diurnal and nocturnal species appear to conserve the same fundamental SCN rhythmicity: electrical activity, neurotransmission, molecular clock mechanisms, melatonin output (signaling the circadian night), and shape of the photic phase response curve are roughly equal in both cases [reviewed in Ref. (70)]. Downstream of the SCN, most hormonal rhythms reflect the changing demands in energy metabolism in coherence with the activity/rest and feeding/fasting cycles (see Figure 2 for an overview of this phase relationships in nocturnal rodents). The same vasopressin SCN signal inhibits (in nocturnal rodents) or stimulates (in diurnal species) adrenal glucocorticoids for mobilizing glucose uptake in brain and muscles (71). Opposite effects for insulin, glycemia, ghrelin, leptin, adiponectin, and lipogenesis/lipolysis have also been reported (6, 72).

As for changes between natural and captivity conditions, while C57/BL6 mice show typical “nocturnal” activity in the laboratory, they were found to be relatively diurnal or even cathemeral in the natural environment (73). However, mice coming from the wild are strictly nocturnal when placed into laboratory conditions (74). This suggests that under artificial LD cycles, the circadian clock is able to manage the entrainment of the activity/rest pattern, which is an essential output reinforcing clock activity, but the temporal activity distribution can also be determined by direct masking of light (75). From the point of view of energy balance, the interaction between LD and temporal niche must optimize food intake, body mass, and energy expenditure. Indeed, locomotor activity can dramatically change to optimize food intake, and/or energy expenditure. In voles exposed to 12:12 h LD with *ad libitum* feeding, access to a running-wheel generates an entrained nocturnal locomotor and feeding pattern, with both SCN and liver clock-genes expression in appropriate phase to the LD cycle. When the voles had restricted access to a running wheel, they showed ultradian activity and feeding bouts every 2–3 h throughout the day and night, with arrhythmicity in liver clock-genes expression, while the SCN remained in phase with the LD cycle (76). A complete temporal niche switching (from nocturnal to diurnal) under 12:12 h LD was observed in mice exposed to cold and simulated food shortage (77). Again, clock-gene expression analysis shows that the underlying neuronal mechanism is downstream from or parallel to the SCN, and that the behavioral phenotype is accompanied by phase adjustment of peripheral tissues. In both examples, temporal niche and peripheral oscillators are phased to reduce metabolic costs: in voles, liver circadian rhythms are suppressed to deal with ultradian digestive demands; in mice, diurnality reduces energy expenditure in a spontaneously nocturnal animal, by reducing thermoregulatory costs, as observed in small burrowing mammals in natural conditions (78).

#### Photoperiod Length

The hypothalamus acts as a photoneuroendocrine integrative center, encoding the photoperiod length at the SCN by changing phase synchronization of electrical activity between individual cells through VIPergic and GABAergic communication [reviewed in Ref. (79)]. In turn, a multisynaptic pathway controls pineal melatonin nocturnal duration by a sympathetic neuronal output (80). Measuring the relative length of the photoperiod (i.e., the number of light hours per 24-h cycle) is a predictive variable for the regulation of energy metabolism in photoperiodic species. For example, the maintenance of high CBT (i.e., euthermia) becomes energetically limited during prolonged periods of cold exposure and food shortages in winter. Seasonal mammals such as the ground squirrel, European hedgehod, or hamsters reduce energy expenditure by entering periods of hypometabolism with temporary suspension of euthermia, to undergo reversible hypothermia (81). As a general view, this state of torpor last less than 24-h and is accompanied by continued foraging. Torpor bouts lasting consecutive days to several weeks occur in hibernator animals that reach lower CBT than daily heterotherms, usually do not forage, but rely on energy stores (82). These animals exhibit annual cycles of adipose tissue accumulation and body weight (83), reflecting changes in the balance between food intake and energy expenditure. The most extended strategy is to increase food intake and body fat in spring/summer, entering hibernation (and bouts of torpor/arousals) during winter by reducing appetite, glycolysis, and catabolizing stored adipose. However, seasonal changes in food intake do not seem to result from hunger/satiety responses to starvation or caloric restriction, neither involving changes in the leptin-responsive pathways in the arcuate nucleus. Although there is a strong modulation of leptin secretion by melatonin (84), the effect of photoperiod on leptin modulation of food intake is still not clear. Plasma levels of adipose tissue hormones leptin and adiponectin respond in proportion to adiposity (85) and to photoperiodic changes in fat deposits. However, increased sensitivity to leptin-dependent mobilization of fat in short-day housed hamsters was not associated with changes in expression in either anorexic or anabolic peptides expressed in leptin-receptor rich structures in the arcuate nucleus (86). Moreover, homeostatic hypothalamic pathways that regulates short-term control of food intake involving mediobasal neuropeptide Y and pro-opiomelanocortin (87), and/or lateral orexin and melanin concentrating hormones [reviewed in Ref. (88, 89)], are likely not involved in this photoperiodic regulation.

When dealing with photoperiod-dependent changes in metabolism, it is particularly important to consider established models of hibernation in the field, including food intake and regulation in hibernating bears (90). While mammalian hibernators cease food consumption for long periods of time, there are profound changes in metabolic sensor pathways that control weight. In particular, grizzly bears exhibit a dramatic change in insulin sensitivity, become insulin-resistant during hibernation but sensitive throughout Spring and Fall (the active periods during which they feed and put on weight) (91). In addition, bears do produce leptin all year-round, but the effect of exogenous administration of the hormone was also different in the Fall (reducing food intake) and the Summer (no effect on food intake). Other hormones also exerted differential effects: ghrelin levels tended to be higher before hibernation, thus contributing to the increase in body mass, and decreased during the winter, in accordance to the low energy utilization and food intake (92). In other words, it is not only the levels of metabolic hormones—e.g., leptin—that change seasonally but also their effects on weight and glucose-sensing mechanisms that might be switched on and off to regulate metabolic rate, food intake, and body weight.

Other hormones are also instrumental in coupling environmental to metabolic pathways. Levels of thyroid hormones are essential for the photoperiodic control of basal metabolic rate and thermogenesis, mainly by sensitizing tissues for the uptake and usage of glucose. Glial tanycytes play a central role in the photoneuroendocrine pathway integrating photoperiod and metabolic state (93). Located in the ependymal layer of the third ventricle in contact with the cerebrospinal fluid, they act as glucosensors that have cellular projections to the median eminence in tight contact with the pars tuberalis of the pituitary gland, and to the arcuate nucleus, regulating their activity. Dense melatonin receptors on the pars tuberalis regulate photoperiodic synthesis and secretion of the thyroid-stimulating hormone β-subunit (TSHβ), which controls tanycyte deiodinase (DIO) expression across a wide range of seasonal mammals (94). During long photoperiods, higher levels of TSHβ and DIO2 favors the conversion of thyroxin (T4) to triiodothyronine (T3), increasing energy expenditure and basal metabolic rate. Lower, short-photoperiod levels of TSHβ promote dominant DIO3 activity, which convert T4 to both inactive reverse T3 and diiodothyronine T2, increasing food intake and adipose deposits (89). In addition, melatonin participates in the phase control of the glucocorticoid rhythm and modulates glucose homeostasis according to photoperiod-dependent metabolic state (95).

#### Melatonin and Metabolism

The importance of melatonin in photoperiodic responses leads to the notion that the pineal hormone is also a key regulator of several metabolic processes. Indeed, early reports revealed that both pinealectomy and the administration of pineal extracts significantly changed glucose levels and utilization in different tissues (96–98). The lack of melatonin can lead to glucose intolerance and insulin resistance (99, 100). Such changes can be reverted by melatonin administration. In particular, melatonin regulates pancreatic function affecting both glucagon and insulin biosynthesis (101, 102). The pineal hormone acts by MT-1 and MT-2 receptor-related mechanisms on the insulin-signaling pathway (103) and, in addition, affects adipose tissue and therefore influences many aspects of metabolism (104–106).

The role of melatonin in the circadian control of energy metabolism has also been demonstrated by several studies that include pinealectomy and melatonin administration or replacement (107, 108). In general, the rhythm in melatonin secretion and plasma levels might influence the so-called “predictive homeostasis,” i.e., the preparation of tissues for optimized responses to internal (e.g., other hormones) and environmental (e.g., diurnal and photoperiodic changes) stimulation. In addition, there are some intriguing results indicating that melatonin might prevent obesity in animal models, presumably through its effect on adipocytes and/or, as indicated above, by affecting thyroid hormones (109, 110).

In summary, the pineal hormone not only couples circadian and seasonal cues to many body functions but might also be a key player in the regulation of metabolic rate. Indeed, changes in melatonin profiles—both experimental and induced by environmental disturbances related to human chronobiological disruptions—might reflect and even produce alterations in metabolism that could be overcome by the appropriate ambient or pharmacological interventions.

#### Light at Night (LAN)

Completely dark nights are set in LD cycles as standard laboratory conditions in most chronobiological studies. However, reducing lighting contrast in LD cycles by dimly illuminated nights demonstrated to be a relevant feature for the circadian clock. The chronic (i.e., more than 1 week) exposure to dim LAN as comparable to moonlight levels (~5 lux), and to levels of night lighting in urban areas, are sufficient to cause important effects on the circadian system of both nocturnal rodents and humans. The effects of LAN were first described at the behavioral level, since it potentiates clock entrainment in Syrian hamsters using different protocols (111–113). Compared to controls exposed to nocturnal darkness, chronic exposure to LAN reduces PER1-2 amplitude at the SCN level, as well as changes in the amplitude of glucocorticoid and melatonin cycles in the Siberian hamster (114). Reduced *Rev-Erb alpha* amplitude, but slight changes in *Bmal1, Cry1-2*, and *Per1-*2 amplitudes were found in murine white adipose and liver tissues, without concomitant changes in both general behavioral activity and glucocorticoid rhythms (115, 116). However, both studies in mice demonstrate that LAN increased body mass and epididymal fat and reduced glucose tolerance. Although daily food consumption was similar to LD controls, LAN reduced the amplitude of the LD feeding rhythm, by increasing food consumption during the light phase (115). The main changes found due to LAN exposure in nocturnal rodents are depicted in the Figure 2.

Since it was shown that mice fed during the day increase their body weight when compared with animals fed *ad libitum* (117), and restricting food access to the night in LAN-exposed mice restores the LD phenotype, this change in the feeding pattern seems to be an important factor for these alterations. Time-restricted feeding (TRF) to the dominant behavioral phase (i.e., night in nocturnal rodents) restoring weight homeostasis was also observed in other protocols (118) and is now being considered an important manipulation to regulate metabolic alterations (119), as discussed below. In addition, placing back the mice in dark nights after exposure to LAN partially reversed the increases in body mass, suggesting that the effects of LAN on metabolism are not permanent (120).

As a completely different approach, it is interesting that artificial nocturnal light might have profound consequences on our ecosystem, not only our physiology. Indeed, a recent study has demonstrated that LAN completely disrupts nocturnal pollination which adds to the expanding list of human activities that threaten ecological relationships in the planet (121).

#### Acute Nocturnal Light Exposure

A single, brief (5–60 min) light-pulse exposure is a typical environmental stimulus used for chronobiological research studying non-parametric photoentrainment (i.e., the acute effects of light on phase-shifting mechanisms of the circadian clock). Moreover, in nocturnal rodents, other behavioral and physiological effects of light are equally important. Acute nocturnal light exposure typically abolishes behavioral activity (i.e., negative masking) for a brief, fixed interval (independent from the stimulus duration but dependent on irradiance) (122). It also induces somnolence and a rapid drop in CBT as well as reduces pineal NAT activity and melatonin synthesis (123, 124), perhaps through a common input pathway to the SCN (125). However, photic masking responses seem to be different between diurnal and nocturnal species (126); for example, such responses persisted after bilateral SCN lesions in the diurnal Nile grass rat (127). It is speculated that locomotor suppression by light in nocturnal rodents is part of the integrative response of the SCN enhancing light effects for the phase-shifting mechanisms (125).

At the peripheral level, the effects of acute nocturnal light on circadian genes has been shown to be fast and directly coupled to metabolic hormonal functions. Light received at ipRGC melanopsin-expressing cells is necessary for the entrainment of corticosterone rhythm to LD (128). Acute LAN processed at the SCN increase corticosterone surge in rats (129) and mice (41) *via* the ANS to rapidly increase *Per1* expression, and also of several genes involved in corticosterone synthesis in adrenal glands (41). Also, exposure to a light pulse at circadian time 15 increases PER1-2 expression in the liver in rats, which is abolished with autonomic denervation (130). Apart from this hierarchical control, using targeted SCN *Bmal1* deletions that preserve neural SCN output circuitry (131), or temporally dissecting the sensitive-specific SCN-photic effects (42), provide intriguing evidence suggesting that light can synchronize peripheral clocks in the kidney, liver, heart, and adrenals, through a retinohypothalamic SCN clock-independent pathway.

### Constant Conditions

The chronic absence of LD cycles is an abnormal condition for most organisms, except those living in extreme latitudes during winter such as the arctic ground squirrel (132) or the reindeer (133). Reindeers suppress their daily activity/rest pattern being active for most of the 24 h during the polar summer day in which constant lighting is present. On the other hand, the constant darkness observed during polar winter day diminished activity bouts and also generates arrhythmicity in reindeer species living at higher latitudes. During the polar Summer day, when constant light occurs, the daily rest/activity patterns of reindeers are suppressed to permit near 24 h active behavior. By contrast, during the polar Winter, constant darkness reduces the activity bouts of reindeers and can lead to arrhythmicity in reindeer living at higher latitudes (but LD rhythms are kept at lower latitudes). Weak circadian (but not photic) control was found for reindeer melatonin levels, as well as for fibroblasts transduced with Bmal1:luc or Per2:luc reporters (134), and is proposed to be a rule particular to polar resident arctic mammals. Still, there are no data on energy metabolism for these species living in such extreme conditions.

#### Constant Light

Chronic exposure to light (LL) is a typical strategy to induce circadian arrhythmicity in nocturnal rodents or to study the circadian free-running period in diurnal species. LL gradually increases the circadian period of motor activity rhythm (in proportion to irradiance) in nocturnal rodents (Aschoff), resulting in arrhythmicity in rat and mice (112, 115, 135). Besides behavioral activity, the circadian feeding and drinking rhythm are also lost under LL (136, 137), and SCN-physiological outputs such as heart rate and CBT also exhibit arrhythmicity (138). Uncoupling of neuronal SCN oscillators (139), altered SCN expression of clock- and clock-controlled genes (135, 140, 141), as well as reduced amplitude of SCN electrical activity rhythms (142) are functional correlates of the circadian behavioral and physiological arrhythmicity. However, *in vivo* imaging of PER2:LUC oscillations in mice under LL showed peripheral rhythms in the liver, kidney, and submandibular glands with decreased amplitudes, and distribution of peak phases irrespective of the state of behavioral rhythmicity (143). This internal desynchronization due to LL is associated with several metabolic disturbances that, to some extent, resemble those observed with dim LAN protocols (see above). In mice, studies have shown that different LL protocols can induce abnormally high levels of body mass and fat tissue, alterations in energy expenditure, develop insulin resistance, and disrupt glucose metabolism, among other problems (115, 142–144). Increased adiposity in mice under LL was also attributed to decreased energy expenditure, by reduced sympathetic β3-adrenergic input to brown adipose tissue for the uptake of fatty acids and glucose for oxidation (145). Studies in rats have reported similar effects, also describing alterations in circulating levels of cholesterol, melatonin, glucose, and corticosterone (137, 146, 147). Figure 2 shows circadian changes under chronic LL exposure in nocturnal rodents, and Table 1 supply references and additional details.

**Table 1:** Abnormal light:dark conditions and metabolic alterations in animal models.

Manipulation	Species	Alterations	Reference
Constant light	Mouse	– Increased body weight – Altered feeding patterns – Reduced glucose tolerance	Fonken et al. (115)

		– Increased body weight – Greater food intake – Decreased energy expenditure – Loss pattern of insulin sensitivity	Coomans et al. (142)

		– Reduced amplitude of liver and kidneys clock-genes rhythms	Hamaguchi et al. (143)

		– Increased fat accumulation in response to high fat diet	Shi et al. (144)

	Rat	– Reduced food and water intake – Increased adiposity	Wideman and Murphy (137)

		– Increased circulating cholesterol levels	Vinogradova et al. (147)

		– Disrupted patterns of plasma melatonin, glucose, lactic acid, and corticosterone	Dauchy et al. (146)

	Hamster	– Altered rhythms of glucocorticoid release	Lilley et al. (148)

Light at night	Mouse	– Increased body weight – Reduced energy expenditure	Borniger et al. (149)

		– Increased body weight	Aubrecht et al. (150)

		– Increased body weight – Altered clock-gene expression in liver and adipose tissue	Fonken et al. (116)

	Rat	– Reduced glucose tolerance	Opperhuizen et al. (151)

Chronic phase shifting	Mouse	– Increased body weight – Increased body fat – Higher levels of triglycerides – Altered adipocytes morphology	Casiraghi et al. (118)

		– Increased body weight – Increased white adipose tissues – Altered liver metabolic genes expression	Oike et al. (152)

		– Altered liver clock-genes expression – Suppression of glucocorticoid and melatonin receptors expression in the liver	Iwamoto et al. (153)

		– Increased progression of colitis	Preuss et al. (154)

		– Increased insulin resistance – Increased fat accumulation	Zhu et al. (155)

	Rat	– Increased body fat – Decreased serum insulin, leptin, and glucose levels – Altered profiles of liver metabolism gene expression	Herrero et al. (156)

		– Increased body weight – Higher fasting glucose levels	McDonald et al. (157)

		– Increased body weight – Reduced activity – Increased food consumption	Tsai et al. (158)

		– Lower plasma insulin levels – Increased fat in response to a high-fat diet	Bartol-Munier et al. (159)

T-cycles	Mouse	– Increased body weight – Increased leptin and insulin levels	Karatsoreos et al. (160)

		– Reduced corticosterone levels	Sollars et al. (161)

Ultradian light cycle	Mouse	– Increased body weight – Reduced activity	Oishi and Higo-Yamamoto (162)

Sleep disruption	Mouse	– Disrupted lipid metabolism gene expression – Increased liver and serum fatty acids	Ferrell and Chiang (163)

		– Altered feeding behavior – Global disruption of liver metabolic transcriptome – Impaired gluconeogenic capacity and glycogen storage rhythms	Barclay et al. (164)

	Rat	– Reduced glucose tolerance	Jha et al. (165)

		– Increased body weight – Altered feeding patterns – Liver clock-genes desynchronization – Disrupted rhythms of plasma glucose and triacylglycerols	Salgado-Delgado et al. (166, 167)

		– Increased body weight	Marti et al. (168)

Mistimed feeding schedules	Mouse	– Increased body weight – Hyperphagia – Induced leptin resistance – Higher levels of plasma insulin – Increased accumulation of cholesterol, triglycerides, and fatty acids in the liver	Yasumoto et al. (169)

		– Increased body weight – Increased calorie intake – Increased respiratory exchange ratio – Altered liver and other peripheral organs clock- and metabolic genes expression – Disrupted daily hormones variations	Bray et al. (170)

	Rat	– Increased body weight – Desynchronization of liver rhythms	Opperhuizen et al. (171)

#### Constant Darkness

Laboratory studies are usually performed under chronic constant darkness conditions (DD) to measure the spontaneous (i.e., free-running) circadian period in nocturnal rodents. In addition, activity/rest patterns in mice and hamsters chronically (i.e., months) exposed to DD are not necessarily stable, evolving in most cases from a consolidated activity bout to fragmented locomotion and eventually into crepuscular bouts (personal observations). Despite this, evidences on regulation of energy metabolism under chronic DD conditions are scant. One study with C57BL/6J mice (172) suggested that DD could be considered a cue triggering metabolic changes for hibernation, since hibernating mammals will seasonally encounter DD conditions inside a den. Although the mouse is not a true hibernator, it can undergo torpor under caloric or thermal restriction, a hibernator-like behavior, entering brief sleep periods lowering CBT down to ~30°C (173). Zhang et al. (172) found that mice exposed to 2 days under DD, or rendered torpid by food deprivation, increased circadian gene expression and activity of fat catabolic enzymes in peripheral metabolic tissues, together with elevated blood 5′-AMP, a typical metabolic switcher acting intracellularly *via* AMP kinase to stimulate fatty acid oxidation, a necessary adaptation when glucose supplies are short. Indeed, a synthetic form of 5′-AMP in LD exposed mice induces the hypothermic torpid state and expression of fat catabolic enzymes. The authors also reported that mice maintained under DD for a week consumed less food and water [similar to rats (174)], decrease body weight, rapidly re-directed metabolic status and showed higher non-esterified fatty acids and lower glucose blood levels compared to mice kept under LD. This is surprising in laboratory mice, which are not usually photoperiodic and are routinely exposed to DD in circadian studies without reported weight loss or metabolic changes. Zhang et al. (172) also reported that peripheral circadian gene expression under DD reverses 5 h after re-exposure to LD, which emphasizes the complex effect of environmental light on peripheral physiology and metabolism. The authors did not assess other indirect effects as putative (acute) increased activity bouts in mice when transferred from LD to DD, increasing energy demand and thus 5′-AMP levels under DD [discussed in: Hastings and Loudon (175)]. In addition, photoperiodic coding by melatonin is absent in C57BL/6J mice, with a mutated gene for serotonin *N*-acetyltransferase (176). Also, it is intriguing that these acute responses to DD found in mice are consistent with how short photoperiods or darkness induce hibernal hypometabolism in photoperiodic species, which are programmed over months (83).

### Chronic Jet-Lag Simulations and T-Cycle Protocols

Protocols of chronic phase-shifting of the 24-h LD cycle (i.e., chronic jet-lag, CJL), or LD schedules with periods different from 24 h (i.e., T-cycles) that affect steady-state entrainment of the SCN clock, demonstrate to be a disruptive condition generating the internal desynchronization of the whole circadian system. It ranges from behavior (177, 178), ventrolateral-dorsomedial uncoupling of *Per1* and *Bmal1* SCN expression (177), to peripheral regulatory outputs as CBT and sleep stages (179) and melatonin secretion (180). Thus, it is not surprising that this chronic misalignment of the circadian clock with the photic cycles leads to metabolic alterations (see Figure 2 and Table 1). Exposing mice to chronic 6-h advances of the LD cycles every 2 days (i.e., CJL by advances) induces weight gain, increases body fat, plasma triglycerides, and adipocyte size, yet without a concomitant increase in food consumption (118). Interestingly, these effects were not observed when applying a similar CJL delaying schedule, which allowed behavioral entrainment. Similar CJL protocols consisting of two 6-h advances on a weekly basis, or of 8-h advances every 2 days also reported disrupted genes expression in the liver (152, 153). Some groups have tested protocols of alternating advances and delays of the LD cycle in rats and found increases in total body weight and adipose tissue along with alterations in metabolic hormones and liver function (156).

Rats exposed to T-cycles of similar length to their endogenous period (T25 and T26) show decreased body weight, food consumption, and less weight gain/food grams when compared with those exposed to shorter T-cycles (181). This experiment provides an interesting hypothesis of circadian-metabolic resonance, in which light cycles very similar to the circadian period efficiently couple SCN-peripheral entrainment to nutrient intake/delivery with energy output. In agreement, mice exposed to shortened T-cycles of 20 h display high body weight, increased leptin, insulin, and insulin/glucose ratio (160). Together, the studies using CJL and T-cycle protocols indicate that chronically forcing the clock to entrain by advances is the worse condition for the body energy metabolism and weight regulation.

## Lighting Environments in Humans

### Photic Responses of the Human Clock

Although social cues also act as relevant zeitgebers, the human circadian clock responds in a canonical way to light, similar to other diurnal mammals. It exhibits the main sensitivity to light in the late biological day/early biological night, delaying up to 3 h the clock phase, a phase advance region (~2-h shifts) in the early biological day, small phase shifts during the middle of the biological day, and a transition point toward the end of the biological night (182). Importantly, this timed response to light is of functional significance for the entrainment of the human clock by natural daylight, shifting its phase (mid-sleep time on free days) to the time of dawn at different latitudes (183). It could be generalized that human rhythms, such as activity/rest and melatonin secretion, become significantly earlier and shift to the natural crepuscules when exposed to strong (“natural”) zeitgebers composed of bright daylight and absence of artificial nightlight (184, 185). Indeed, light pollution in urban areas, i.e., the artificial lighting during the biological night, might have induced a shift of human activity toward the night and is emerging as a negative factor for health increasing the risk for metabolic disorders (186). Altered exposure to the natural LD cycle by attenuated exposure to daylight within buildings or increased exposure to artificial nightlight is one of the mechanisms leading to insufficient sleep (187). This is the so-called social jet-lag, i.e., the situation in which the body’s biological clock and the actual sleep schedules do not match, and which can be quantified as the difference between mid-sleep time on work days and mid-sleep time on free days (188). Poor (self-reported) lighting exposure, together with metabolic factors (changes in weight and appetite) of the seasonal variations in mood, is a key to the MS in the Finland population (189). In addition, irregular activity-rest patterns with higher interdaily stability was also associated in older (81.6 ± 7.5 years) adults with MS (190), although photic effects on the daily patterns were not determined in this study.

Light intensity of ~200 lux is able to shift the human circadian clock (191) and even very dim light can decrease plasma melatonin levels (192), which indicates that subjects under typical indoor illumination can receive enough irradiance at night to affect their circadian system. Higher levels of LAN exposure (>3 lux) in elder people, resulted in higher body weight and body mass index (BMI), elevated triglyceride, and LDL-cholesterol levels independent of demographic and socioeconomic parameters, and without changing melatonin levels (193). Using satellite images of night time illumination, and clinical data on countries worldwide, a statistical significant association has been found between the exposure to LAN and being overweight (194). A similar study in Korean urban areas also found this association together with delayed and disrupted sleep (195). Taking together, the data from Wright et al. (184), de la Iglesia et al. (185), and Roenneberg and Merrow (188), it could be hypothesized that humans living in urban areas with access to electrical light likely experience LAN, delaying its biological clock, and therefore generating social jet-lag. Remarkably, a large-scale epidemiological study has demonstrated that social jet-lag is a key factor increasing BMI and obesity, independently of sleep duration (196). Greater social jet-lag associated positively with triglycerides, fasting insulin, insulin resistance, waist circumference, and BMI, and negatively with HDL cholesterol (197).

Our current 24-h society requires an increasing number of employees to work nightshifts and, as a result, millions of people worldwide work during the evening or night for a certain period of their life. Most epidemiological studies indicate that LAN is a key factor in shift workers to develop metabolic impairments (198), and the risk of MS increases in health-care personnel working night shifts (199). However, the establishment of a direct link between LAN affecting the circadian clock and metabolic disease in shiftworkers has been prevented by a lack of consistent, quantitative methods for measuring LAN supplying contradictory results [reviewed in Ref. (200)]. Moreover, nightworkers switch their temporal niche by a 180° shift of the activity/rest rhythm, imposing a circadian misalignment in the timing of energy metabolism for a diurnal mammal. The SCN clock tends to entrain to the LD cycle driving endocrine and autonomic outputs for diurnality, as for instance peaking insulin sensitivity at noon in subcutaneous adipose tissue (201). Night work forces the circadian system for repetitive arousals during the night, to sleep during the day, and to eat at abnormal circadian times. To simulate circadian misalignment due to shiftwork in laboratory conditions, imposing an activity/rest pattern different from 24 h during 7 days generates eating and sleeping ~12 h out of phase from habitual times (202). This misalignment systematically decreases leptin, increases glucose despite increased insulin, increases postprandial glucose, completely reverses the daily cortisol rhythm, increases blood pressure, and reduces sleep efficiency. Similar studies also showed decreased insulin sensitivity (without insulin increase) (203) and altered rhythm of energy expenditure (168). This forced desynchronization protocols showed how changing the feeding pattern and timing of metabolic signals for only a week, leads to cardiometabolic alterations and a prediabetic state. In a 3-day study of 12-h simulated shiftwork, the misalignment between the endogenous circadian system and daily environmental/behavioral rhythms with nighttime feeding, affected glucose tolerance, by elevating postprandial glucose level. These changes in glucose metabolism are related to different insulin mechanisms: a decreased pancreatic β-cell function and decreased insulin sensitivity (204). Sleep deprivation and melatonin disruption are also observed in subjects chronically subjected to shiftwork (205), where sleeping less than 6 h daily was associated with weight gain and an increase in BMI (206).

Shiftwork exposure has been hypothesized to increase the risk of chronic diseases, including cancer, cardiovascular disease, MS, and diabetes (198). Several epidemiological studies have evaluated the association between shiftwork and MS. This syndrome is a cluster of risk factors including central obesity, elevated blood pressure, elevated triglycerides, lowered high-density lipoprotein cholesterol, and elevated fasting glucose, which are often seen simultaneously in an individual, and is a risk factor for cardiovascular diseases. Table 2 summarizes information regarding the association between night shiftwork and the different parameters of this syndrome. In addition, these data indicate an association of shiftwork schedules with low concentrations of HDL cholesterol, higher diastolic and/or systolic arterial blood pressure, fasting glucose, insulin, and/or triglycerides. Some of these reports also show an increased risk factor of MS associated with a longer period of time working at night or in rotating schedules (207, 208). Moreover, these associations are stronger in females than in males (207, 209), and indeed it has recently been shown that the risk of obesity in shift workers was not related to these factors in males (210).

**Table 2:** MS symptoms in people working at night or in rotating shift.

*n*	Population	Age (years)	Gender (F/M)	Country	Years of shiftwork	Schedule	Hours worked per shift/started at	Alteraciones	Reference
27,485	nd	30–50 years	F/M	Sweden	nd	Self-reported	nd	Obesity, high triglycerides, and low HDL cholesterol	Karlsson et al. (211)
437 DW, 246 SW	Factory employers	34–38 years	M	Argentina (European Ancestry)	nd	4 days, 3 rest, 2 nights, 3 rest, 4 nights, 3 rest, 2 days, 3 rest	12 h/06:00 h and 18:00 h	High waist-hip ratio, diastolic arterial blood pressure, fasting glucose and insulin, HOMA index, and triglycerides	Sookoian (212)
98 DW, 100 SW	Petrochemical plant employers	39–60 years	M	France	>10 years	1–2 days, 1–2 afternoon, 12 night, 3–4 rest	8h/05:00, 13:00, and 21:00 h for SW, 08:00 for DW	Rise triglycerides, free fatty acids, and gGTP and lower HDL cholesterol	Esquirol et al. (213)
1220 DW, 309 SW	Employers of public administrations, private companies and a bank	35–59 years	M	Belgian	>20 years	Two rotating shifts	nd	High body mass index (BMI), waist circumference, systolic and diastolic blood pressure, low HDL cholesterol	De Bacquer et al. (214)
336 DW, 402 SW	Nurses	37–40 years	F/M	nd	>1 year	4 nights per month	nd	High BMI. Predictors of MS: sedentariness and SW (4-year follow-up: 9 vs 1.8%)	Pietroiusti et al. (199)
125 DW, 165 eSW, 102 pSW	Workers of an electronic manufacturing company	25–31 years	F	Taiwanese	>5 years	6 days, 3 rest, 6 night, 3 rest	12 h/07:30 h and 19:30 h	Increased percentage of development of MS in pRSW (5 years follow-up). Obesity and elevated blood pressure in pRSW	Lin et al. (215)
26,382	Dongfeng Motor Corporation’s employers	Retired workers (average 63.6 years)	F/M	Chinese	1–10, 11–19, or >20 years	2 shifts, 3 shifts or 4 shifts	12, 8, and 6 h, respectively	Long-term shift work associated with MS in females. High blood pressure, waist circumference and glucose levels	Guo et al. (207)
2,661 DW, 656 SW	nd	20–40 years	F/M	South Korean	nd	Self-reported	nd	Shift work was associated with MS in females	Yu et al. (209)
370 DW, 354 SW	Nurses and midwives	40–60 years	F	Poland	<10, 10–20, or >20 years	2–7, or >8 night per month	12 h/7 p.m.	Increased BMI, waist circumference and obesity in women reporting >8 night shifts per month	Pietroiusti et al. (199)

*MS, metabolic syndrome; DW, day workers; NW, night workers; SW, rotating shift workers; eSW, ever rotating shiftwork; pSW, persistent SW*.

A dysregulation in the mechanism of hunger/satiety control and in energy expenditure may be related to the increase of BMI associated with night shift work. First, meal distribution during shiftwork is different. Shift workers tend to have smaller meals for breakfast and lunch but increase their snacking behavior. In the afternoon or nighttime, the frequency of snacking increases, with a tendency for a higher consumption of carbohydrates (199), which is indicative of obesity-related eating behaviors (213, 216). Second, a study including 14 adults in a 6-day simulated shiftwork protocol, showed decreased total daily energy expenditure in nightshift days compared with baseline, driven predominantly by a decrease during the sleep time (217). Third, there was an increase of fat utilization, together with a decrease of carbohydrate and protein, with similar levels of the hunger-stimulating hormone ghrelin and decreased levels of the satiety hormones leptin and peptide YY.

### Other Protocols of Circadian/Metabolic Disruption

While light is the most potent zeitgeber, the availability of non-photic cues such as food and exercise, may alter the internal circadian clock independently. Instead of modifying the environmental cues, researchers force the timing of otherwise naturally entrained behaviors or physiological variables. Two simple behavioral interventions are to manipulate the timing of wake/sleep and to schedule feeding times. These behaviors represent both controlled outputs of the clock and feedback mechanisms or inputs to the system. Because of this, altering the phase of such events in relation to the light cycle (like when humans work night shifts) can have a profound effect on biological rhythms and hence in metabolism (see Table 2).

Sleep deprivation in mice has profound consequences on liver activity from the gene transcription level to metabolism of fatty acids and other metabolites and also alters feeding patterns (218, 219). Instead of simply preventing sleep, researchers have developed a “forced work” protocol to keep rats awake by forcing them to remain active by placing them in constantly rotating running wheels. Under this protocol, rats develop obesity, show altered feeding behavior, and glucose and triglycerides levels, as well as desynchronization of clock-genes expression in the liver (166, 167).

Timing of food intake affects the rhythms of clock-gene expression in the liver (37, 39), the dorsomedial hypothalamus and the paraventricular nuclei, and the pituitary gland tissues, but not in the SCN (220). In consequence, feeding during the “wrong time” (e.g., the resting light phase in nocturnal animals) can internally desynchronize the circadian system, leading to disease. Studies have shown that forcing mice or rats to feed during the light phase leads to increased body weight and food consumption, variations in hormonal and metabolite levels in plasma, fat accumulation, alterations in the liver, and MS (169–171, 220, 221).

### Strategies to Overcome Metabolic Effects of Circadian Disruption

The unconstrained capacity to manipulate housing lighting conditions in the laboratory might be considered a prerequisite to test potential therapeutic tools. For that reason, researchers have sought model animals to develop strategies that may either overcome or alleviate the symptoms associated with situations of disruption of the circadian-metabolic system.

Behavioral interventions appear as easy alternatives in this sense and represent translatable potential tools for further research in human populations. The main objective of such protocols is either to reinforce entrainment to the LD or other experimental cycle or to provide a different entraining cue that may reorganize rhythms in a way to avoid possible alterations. Scheduling feeding regimes can be as deleterious when “wrongly” timed as can be beneficial under certain challenging conditions. Many studies have shown that TRF can be beneficial, for example, to counter the effects of obesity-inducing diets (222, 223). But it also can help to combat the consequences of protocols that disrupt the biological rhythms and metabolism.

Time-restricted feeding to a favorable circadian timing, i.e., when caloric intake can manage to balance anabolic and catabolic processes with energy expenditure, represents a therapeutic (and experimental) tool preventing metabolic alterations leading to weight homeostasis alterations (119). Some of the reported benefits in rodents include improved glucose tolerance, reduced triglyceride, reduced cholesterol, reduced systemic inflammation, and improved endurance. Forced-desynchronized mice under CJL can keep control weight values when fed only during the dark phases, with no changes in food intake (118). Rats that gain weight under the previously described “forced work” protocol during the rest phase can be phenotypically rescued by preventing feeding during that period and allowing it during the dark phase (166). TRF can be such a strong key that is able to reinstate the *Rev-erb*α and *Bmal1* (but not *Per1-2*) gene expression rhythms at the SCN in behaviorally arrhythmic mice under LL (141).

Wehrens et al. measured the effect of a 5-h meal delay on the human circadian system and observed that late meals delay rhythms of plasma glucose and adipose PER2 clock-gene expression, suggesting that meal timing may help to reset the circadian system in cases of shift work (224). A 16-year longitudinal study reported a 27% higher risk of coronary heart disease (measured by BMI, hypertension, hypercholesterolemia, and diabetes) in men who skipped breakfast and a 55% higher risk in men who ate late at night, in comparison with men who eat normally. However, no association was observed between eating frequency (times/day) and risk of coronary heart disease (225). In addition, a recent study using a smartphone app to monitor eating time in healthy adults revealed a daily eating pattern highly variable from day to day, indicating that more than 50% of adults spread their daily caloric intake over 15 h or longer. Interestingly, this report showed that reducing the daily eating duration to 10–11 h for 16 weeks, without decreasing calories or change nutrition quality, induced weight loss and improved sleep (226). This observation, together with previously mentioned data recorded in animal models, suggests an important role of eating patterns in metabolic diseases.

Running-wheel activity has been shown to represent a strong feedback mechanism to the circadian system, and as such represents a potential therapeutic tool for chronodisruption models. Voluntary wheel running enlarges the boundaries of behavioral entrainment to T-cycles (227) and increases the amplitude of SCN electrical activity rhythm (228). It was found that wheel access in mice modulates CBT and heart rate rhythm waveforms (i.e., increase these variables during or after energy demand for exercise) and couples the PER2:luc phase of adrenal and liver oscillators driving cardiometabolic output (229). In this direction, we demonstrated that the availability of a running wheel allowed for correct entrainment and prevented the elevated body weight gain observed in mice under the desynchronizing CJL schedule. We also found that social contact, achieved by housing mice in groups, is a successful intervention to alleviate metabolic symptoms in this model (118).

Of course, pharmacological tools have also been tested. One approach is to try to replace or substitute the action of disrupted hormonal function. For example, melatonin administration during the subjective night prevents MS-like symptoms in rats under LL (147). Another study showed that metabolic alterations derived from CJL protocols were prevented by treatment with estrogens (155). Yet, another type of pharmacological approach that requires further research is the application of drugs with the ability to induce or reinforce entrainment to environmental cycles and its changes.

## Discussion

### Potential Pathways Linking Photic Environmental Challenges to Metabolic Disease

Protocols with photic alterations in nocturnal rodents (such as LAN and LL) provide evidences to approximate metabolic alterations due to circadian disruptions elicited by nocturnal light pollution in humans. Receiving LAN appears to be a common disrupting factor for both nocturnal and diurnals. The main limitations for these approaches, however, are that temporal niche determines a 180° difference between nocturnal rodents and humans, also regarding the regulation of metabolic variables and energy expenditure for behavioral activity. In addition, nocturnal rodents are more sensitive to nocturnal light than humans. Behavioral and feeding pattern misalignment with lighting schedule seems to be a key factor for most alterations, as discussed below.

Endocrine pathways directly modulated by light can play a role in photic-related metabolic alterations. Melatonin is released by the pineal gland and its nocturnal production is sensitive to light exposure during the circadian night (230). Its roles in metabolism go from participating in insulin production and secretion, synchronizing feeding with the organism’s optimal metabolic state [reviewed in Ref. (108)], and regulation of leptin rhythm (95) photoperiodic resistance (see above). Also, melatonin rhythm disruption correlates with that of adipokines and obesity, and its exogenous administration reverse this phenotype (84). Because of this, it is tempting to speculate that disruption of the melatonin pathway under challenging light conditions (see Table 1) may underlie metabolic dysfunction and obesity. Indeed, LL and LAN protocols could be integrated by the SCN and melatonin signal as a “lengthened” photoperiod, thus encoding a summer-like metabolic state, increasing food intake and adipose deposits (see above). However, there is evidence that animals lacking melatonin still suffer LAN-induced metabolic alterations and loss of weight homeostasis (115, 144), and the link of melatonin with leptin in human obesity is still inconsistent (84). Thus, photic melatonin disruption *per se* does not appear to be a factor linked to obesity neither in nocturnal rodents nor in humans.

Glucocorticoid hormones are key to the regulation of metabolism and are also tightly linked to the circadian system (231), and as such, can also be considered as an underlying cause for metabolic disease in conditions of biological rhythms disruption. Alterations in the 24-h rhythm of glucocorticoid levels may promote circadian misalignment and its adverse health consequences. Multiple studies have shown that the 24-h profile of cortisol concentrations does not adapt rapidly to acute shifts in light–dark, activity–rest, and/or feeding cycles, as occurs in jet lag and shift work rotations, although its amplitude may be reduced under some conditions (232). But the revision of the literature on animal models (67) and human subjects summarized in the previous sections shows that under the different disrupting light conditions, glucocorticoids are impaired, increased, or not affected at all. A study in humans has found that light effects on cortisol are strongly dependent on light intensity, which can explain the seemingly contradictory reports in both humans and laboratory animals (233).

Figure 2 depicts a scheme of the main circadian changes found using protocols of photic desynchronization in nocturnal rodent models (mice and rats), to help the construction of a generalized framework. It seems that photic hormonal pathways alterations *per se* cannot be considered the sole triggers of metabolic disturbances under disrupting lighting conditions. Photic inputs to the SCN can directly and indirectly affect physiological responses given the presence of multiple pathways regulating peripheral oscillators (see above). Importantly, peripheral synchronization for metabolic organs such as the liver is achieved mainly by SCN entrainment of the feeding/fasting pattern, and derived entraining signals can act indirectly of photic effects (234) through blood-borne nutrients assimilated and/or metabolites (3). In this sense, a desynchronized feeding/fasting pattern with temporal niche driven by LD cycles can be considered as a main factor for most metabolic alterations.

### Desynchronization of Feeding Patterns

We will now focus on behavioral effects of light disruption that have also metabolic implications: meal time shift, activity levels, and sleep disruption. To distribute feed bouts or meals during the rest phase, tending to the abolishment of the feeding/fasting pattern, results a common feature of most the aforementioned lighting protocols in rodents (dim LAN, LL, or CJL) (see Figure 2). Indeed, most metabolic disruptions are reversed by scheduled feeding (see Strategies to Overcome Metabolic Effects of Circadian Disruption), restoring normal weight homeostasis under CJL, even while gross locomotor activity remains desynchronized with the LD cycle (118). The same can be said of decreased or disrupted locomotor activity levels and rhythms, or even the sleep/wake pattern (235, 236).

Postprandial glycemia is under circadian control (71). In humans, there is a decreased glucose tolerance during the evening and night as result of a reduced insulin secretion, as well as peripheral and hepatic insulin resistance. This pattern is independent of both the sleep/wake and feeding/fasting rhythms (204, 237) but depends on glucocorticoid rhythm (238). Moreover, increased caloric intake when the body is not able to manage it (i.e., to storage, mobilize, or fuel) is a key factor to develop insulin resistance, MS, and obesity (239, 240).

**Figure 3:**
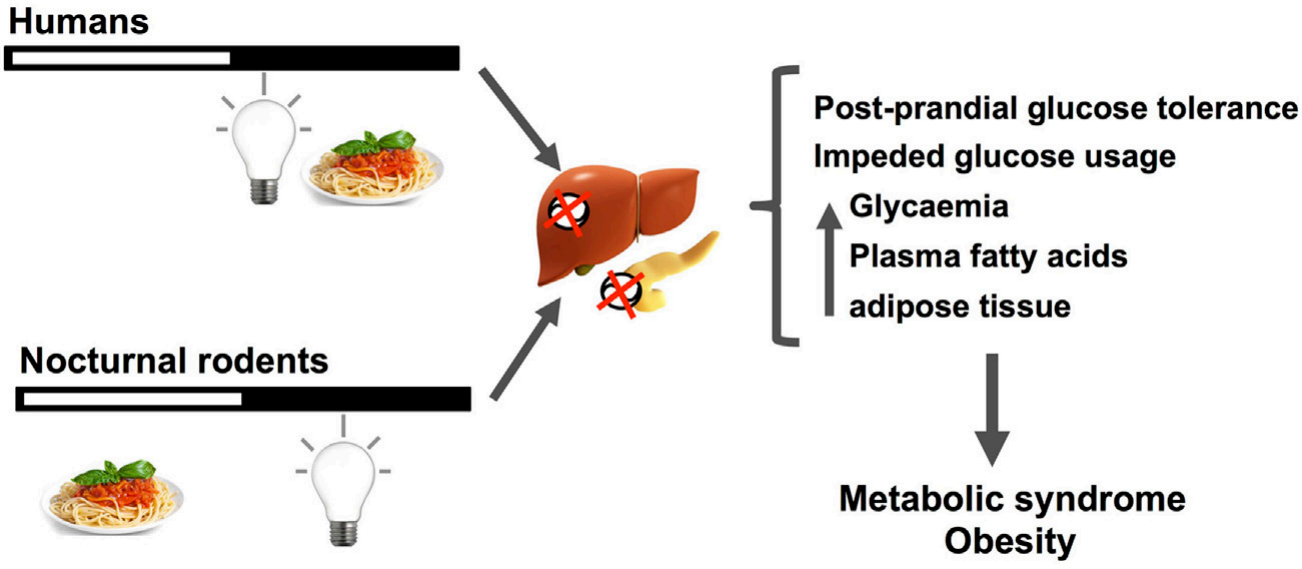
The figure schematizes how nocturnal light exposure, together with a misaligned feeding pattern with respect to the regular activity/rest rhythm, generate metabolic disruptions in nocturnal rodents and human subjects. Humans under nightwork schedules tend to invert by 180° their activity/rest rhythm, increasing nocturnal feeding and light exposure during the night. When receiving light at night, nocturnal rodents alter their feeding pattern increasing episodes throughout the day. These changes desynchronize pancreatic and liver circadian functions regulating nutrient balance and caloric usage/storage, mainly by reducing postprandial glucose tolerance, and decreasing glucose usage. As main outcomes of these alterations, increased basal glycemia, free fatty acids, and adipose tissue are generally observed. When chronically established, this misalignment between the circadian clock activity, its photic inputs, and behavioral/physiological/metabolic outputs can lead to metabolic syndrome and obesity.

Circadian misalignment due to behavioral and/or photic desynchronization (e.g., shiftwork or CJL) alters endocrine and autonomic pathways set to manage metabolic fuels needed for energy demand (i.e., glucose, fatty acids, or ketone bodies under chronic starvation). Figure 3 schematizes the main disruptions that can be generalized in nocturnal rodents and nightworkers. First, the daily amount of food intake seems to be the same among day and shiftworkers. Wrongly timed circadian feeding leads to postprandial glucose increase, first due to misaligned circadian glucose sensitivity at the pancreas, both in nocturnal rodents (6) and humans (202, 204). Importantly, eating at night during simulated shiftwork reduced total daily energy expenditure during nightshift schedules (217). Thus, impairments in glucose uptake (i.e., a prediabetic state) can be chronically established due to changing the set point for glycemia, leading to further insulin insensitivity, and hyperglycemia. It is conceivable that, starting from reduced nighttime postprandial glucose tolerance, chronic dysregulation in the uptake and/or usage of glucose can lead to further increased usage of fatty acids from triglycerides stores in the liver and adipose tissue. This misbalance between carbohydrates/triglycerides usage can lead to dyslipidemia and hyper-triglyceridemia (i.e., increased free fatty acid levels) found in most animal and human studies described above and increased caloric storage. In addition, these alterations in caloric intake and storage, where also attributed to negative energy balance imposed by sleep debt (217, 241). Thus, eating, and being awake at the wrong time, i.e., when the circadian clock promotes sleep, is a risk factor for MS, weight gain, and obesity.

## Conclusion

We have covered a vast amount of evidence from laboratory rodent models and human studies of the diverse effects of lighting environments challenging biological rhythmicity on metabolism. While there are several features and alterations which repeatedly appear under the environmental conditions studied, no single pathway capable of explaining the disrupted metabolic can be clearly identified. We conclude that no particular alteration can be considered as the unique determinant pathway for metabolic disease caused by abnormal lighting protocols. Our hypothesis considers an accumulation of different disturbances at various levels, adding up to a global metabolic disruption, and further leading to disease. The combination and the level of specific alterations will depend on the features of circadian disruption, which is tied to the specific protocol, be it LL, LAN, or CJL. Circadian disruption, including photic effects on the circadian physiology, forced temporal niche shifting of behavioral and feeding patterns (i.e., by LAN, or shiftworking) could therefore represent the fundamental origin of metabolic alterations under abnormal lighting conditions. Although a complex scenario, to better understand how a pathological metabolic state occurs, we must first determine which pathways drive and integrate the interactions between circadian and metabolic homeostasis.

## Author Contributions

SP, LC, PM, NP, DG, and JC had made substantial contributions for this work, contributing with the reviewed data and its interpretation in all the sections of the manuscript, drafting the work and revising it critically, approving the final version, and agreeing for all aspects of the word in ensuring that questions related to the accuracy or integrity of any part of the work were appropriately investigated and resolved.

## Conflict of Interest Statement

The authors declare that the research was conducted in the absence of any commercial or financial relationships that could be construed as a potential conflict of interest.
